# Herpes Simplex Virus Type-1 Infection as a Trigger for Pemphigus Vulgaris

**DOI:** 10.7759/cureus.89219

**Published:** 2025-08-01

**Authors:** Ana Toste, Humberto Machado, João C Almeida, José M Lopes, José Costa

**Affiliations:** 1 Internal Medicine Department, Unidade Local de Saúde de São João, Porto, PRT; 2 Medicine, University of Porto, Porto, PRT; 3 Intensive Care Medicine Department, Unidade Local de Saúde de São João, Porto, PRT; 4 Pathology Department, Unidade Local de Saúde de São João, Porto, PRT; 5 Institute of Molecular Pathology and Immunology of University of Porto (IPATIMUP) and Institute for Research and Innovation in Health (i3S), University of Porto, Porto, PRT; 6 Internal Medicine Department, Hospital da Luz Aveiro, Aveiro, PRT

**Keywords:** auto-immunity, hsv-1 infection, oral ulcers, pemphigus vulgaris, rituximab

## Abstract

Pemphigus vulgaris (PV) is a mucocutaneous autoimmune disorder that can be challenging to diagnose and treat. We discuss a clinical case of a patient with a previous medical history of autoimmune gastritis and vitiligo who presented with painful oral ulceration but was devoid of dermatological manifestations. Immunoglobulin M (IgM) antibodies and polymerase chain reaction (PCR) amplification of Herpes simplex virus type 1 (HSV-1) were detected from the base of the ulcers. Treatment with acyclovir only modestly improved the lesions. Anti-desmosome antibodies were positive, and an oral biopsy showed PV features. Treatment with prednisolone followed by rituximab (RTX) resulted in significant improvement.

## Introduction

Pemphigus vulgaris (PV) is a rare and devastating autoimmune disease of the skin and mucous membranes. It presents with flaccid bullae and erosions in the skin and mucosa [1]. The condition can be life-threatening, with reported mortality rates ranging from 5 to 15% [2], often as a result of infectious complications. Patients with pre-existing autoimmune diseases are at an increased risk of developing PV. PV is an antibody-mediated disease in which antibodies against desmogleins 1 and 3 attack the epitope structure of desmosomes, triggering a type II hypersensitivity reaction leading to the destruction of cell surface receptors, thereby compromising intraepidermal adhesion [3,4].

A biopsy demonstrating immune-mediated desmosomal breakdown and loss of intercellular integrity among keratinocytes above the basement membrane, a phenomenon known as intraepidermal acantholysis [4,5], is necessary to establish the diagnosis. The treatment can be challenging. The core of the treatment is glucocorticoids, followed by other immunosuppressive agents, such as azathioprine, mycophenolate, and cyclophosphamide [5]. Rituximab (RTX) has emerged as a novel and promising treatment option for PV, especially in refractory cases [6,7].

## Case presentation

A 48-year-old Caucasian female with a medical history of autoimmune gastritis, vitiligo, and pernicious anemia (managed with parenteral cobalamin supplementation) presented to the emergency department (ED) of Unidade Local de Saúde de Santo António with a two-day history of painful oral ulceration. She did not report any skin lesions or systemic symptoms. Hemorrhagic blistering and aphthous ulcerations were observed on physical examination. Blood tests, including complete blood count and coagulation studies, were unremarkable. The patient was treated with sucralfate and topical lidocaine for pain relief. Three days later, she was observed at a private care facility for the same complaints and medicated with amoxicillin 875 mg and clavulanate potassium 125 mg per os (PO) twice daily for seven days and deflazacort 30 mg PO twice daily for five days with partial improvement.

Two weeks after the first medical assessment, the patient sought care at the ED of our hospital, Unidade Local de Saúde de São João, for recurrence of painful blistering and bleeding ulcers, this time associated with aphonia, odynophagia, asthenia, and 7 kg weight loss. Upon physical examination, she was found to be afebrile and hemodynamically stable. Moderate dysphonia was noted. Auscultation of the lungs and heart was unremarkable, and there were no palpable lymphadenopathies or skin lesions besides patchy discoloration of the hands that were compatible with vitiligo. she was referred to Stomatology and Otorhinolaryngology (ORL) for observation, where both evaluations documented extensive ulceration of the jugal mucosa, oral pavement, ventral and lateral regions of the tongue, soft palate, and epiglottis, as well as arytenoid edema and erythema. The ulcers displayed sharp, irregular borders. Nikolsky's sign was unclear. Laboratory results were relevant for leukocytosis and mild C-reactive protein (CRP) elevation. HIV and HCV serologies were negative, and she had vaccine immunity for HBV. She was started on prednisolone 40 mg PO once daily for seven days, after which she was reassessed at the Stomatology Clinic. She showed overall improvement in the lesions, and a tapering regimen of glucocorticoids was initiated.

**Figure 1:**
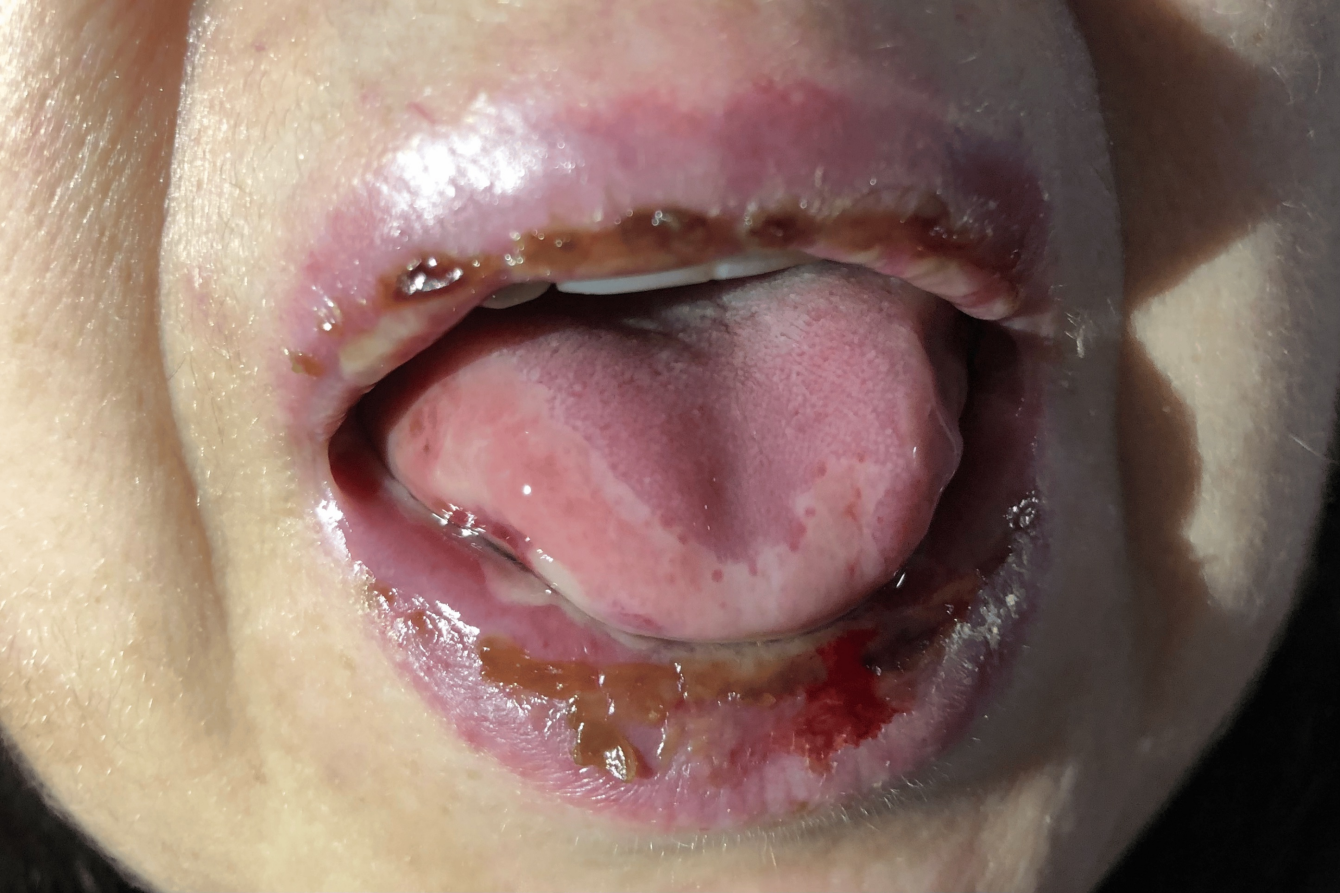
The patient's mouth and tongue at the time of admission to the Internal Medicine infirmary displaying extensive ulceration of oral mucosa

One week after initiation of glucocorticoid tapering, the patient returned to the ED of our hospital for malaise, asthenia, worsening of the oral lesions, and feeding difficulty. Physical examination was remarkable for fever and worsening of the oral ulcers, which exhibited purulent exudate and a fetid odor (Figure 1).

Laboratory assessment revealed worsening leukocytosis and increasing levels of CRP. Blood cultures were obtained, and the patient was admitted to the Internal Medicine infirmary for treatment and further study. Treatment with prednisolone 10 mg, in line with the ongoing tapering scheme, was maintained. For the following 24 hours, she exhibited sustained fever and worsening of inflammatory markers. Then, she was started on empiric antibiotic treatment for infected oral ulcers with piperacillin-tazobactam 4.5 mg intravenous (IV) every eight hours. The diagnostic workup is summarized in Table 1.

**Table 1:** Blood, urine, and oral swab test results upon the patient's admission to the infirmary MCV: mean corpuscular volume; MCH: mean corpuscular hemoglobin; ALT: aspartate transaminase; ALT: alanine aminotransferase; GGT: gamma-glutamyltransferase; CMV: cytomegalovirus; EBV: Epstein–Barr vírus; HSV: herpes simplex virus; IgG: immunoglobulin G; IgM: immunoglobulin M; TPPA: treponema pallidum particle agglutination assay; QN: quantitative; PCR: polymerase chain reaction

Test	Result	Normal range
Red blood cells (x 10^12^/L)	4.19	4.0-5.0
Hemoglobin (g/dL)	12.2	12.0-16.0
Hematocrit (%)	38.3	37-49
MCV (fL)	91.4	87-103
MCH (pg)	29.1	27-35
White blood cells (x 10^9^/L)	11.95	4.0-11.0
Neutrophils (%)	70.7	53.8-69.8
Eosinophils (%)	6.4	0.6-4.6
Basophils (%)	0.2	0.0-1.5
Lymphocytes (%)	14.1	22.6-36.6
Monocytes (%)	8.2	4.7-8.7
Platelet count (x10^9^/L)	240	150-400
Plasma proteins (g/dL)	47.5	64.0-83.0
Albumin (g/dL)	25.6	38.0-51.0
AST (U/L)	10	10-31
ALT (U/L)	10	10-31
GGT (U/L)	9	10-32
Alkaline phosphatase (U/L)	74	30-120
Bilirubin, total (mg/dL)	0.45	<1.20
Lactate dehydrogenase (U/L)	155	135-225
Creatine kinase (U/L)	33	10-149
Urea (mg/dL)	12	10-50
Creatinine (mg/dL)	0.75	0.51-0.95
Creatinine clearance (CKD-EPI) (ml/min/1.73m2)	94	90-125
Sodium (mEq/L)	141	135-147
Potassium (mEq/L)	3.2	3.5-5.1
Chloride (mEq/L)	104	101-109
C-reactive Protein (mg/L)	202	<3.0
Procalcitonin (ng/mL)	0.08	<0.05
CMV IgM	Negative	
CMV IgG	Positive	
EBV IgM	Negative	
EBV IgG (Early)	Negative	
EBV IgG (EBNA)	Positive	
EBV IgG (VCA)	Positive	
HSV-1 IgM	Positive	
HSV-1 IgG	Positive	
HSV-2 IgM	Negative	
HSV-2 IgG	Negative	
TPPA	Negative	
EBV DNA, QN PCR (whole blood)	Not detected	
CMV DNA, QN PCR (whole blood)	Not detected	
HSV-1 DNA, PCR (oral swab)	Detected	
Blood culture test (2 samples)	Negative	
Urine culture test	Negative	
Anti-desmosome antibodies	Positive	
Immunoglobulin G (mg/dL)	1050	650-1500
Immunoglobulin A (mg/dL)	359	78-312
Immunoglobulin M (mg/dL)	146	55-300

Serologies for Herpes, cytomegalovirus, and Epstein-Barr virus were obtained, as well as blood PCR amplification tests for cytomegalovirus and Epstein-Barr virus. Serologies were positive for IgM and IgG antibodies for Herpes simplex virus type 1 (HSV-1), suggesting acute infection. A swab of the base of the oral ulcers was sent for a PCR test and was positive for HSV-1, confirming HSV-1 oral infection. Treatment with acyclovir 5 mg/kg IV every 8 hours was added to the antimicrobial regimen. The body CT scan showed no signs of malignancy, making the differential diagnosis of paraneoplastic syndrome less likely. Other sites of infection were excluded as the source of elevated inflammatory markers. A stomatology evaluation was procured, and a biopsy of the mucosal ulcers was performed. Anti-desmosome antibodies were measured.

The patient was discharged after the completion of a seven-day treatment course of piperacillin/tazobactam. There was an overall improvement in the health status. She was afebrile, oral pain was controlled, and she was able to have adequate oral intake. The ulcers no longer displayed purulent exudate and showed signs of impending cicatrization. She was discharged on acyclovir 400 mg PO every eight hours to complete a 10-day treatment course, colchicine 0.5 mg PO every 12 hours, and prednisolone 10 mg PO for one week, followed by tapering. Results from oral biopsy and anti-desmosome antibodies were pending at discharge. Two weeks after discharge, she was reassessed at the Internal Medicine clinic. She had completed prednisolone tapering and was only medicated with colchicine. She reported three small erythematous ulcers in the tongue and jugal mucosa, with no signs of bacterial superinfection. Anti-desmosome antibodies were positive. Oral biopsy results were still pending. Treatment with prednisolone 10 mg daily was reintroduced, with a presumptive diagnosis of PV.

**Figure 2:**
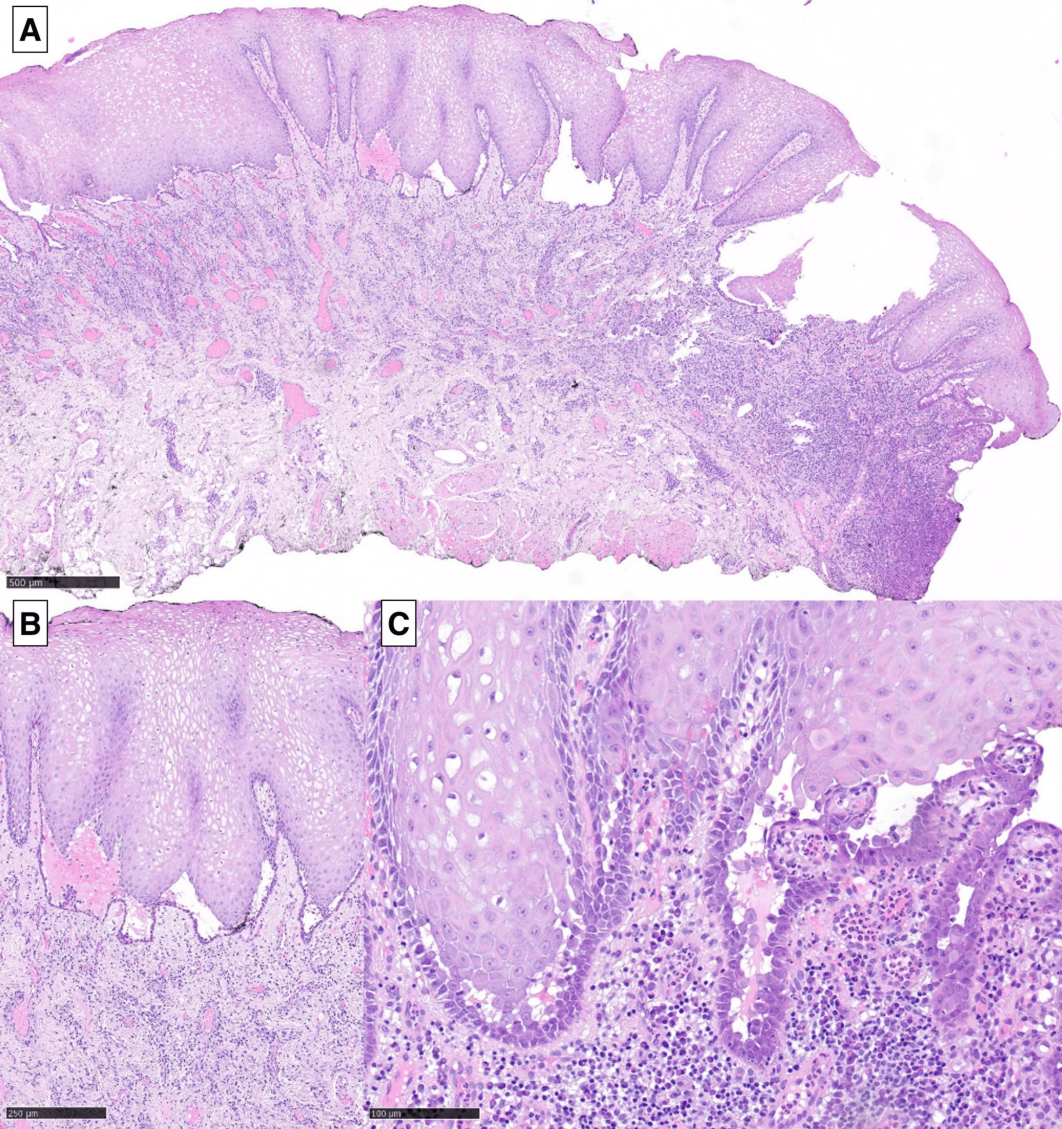
Histopathological features of the patient's jugal mucosa biopsy showing characteristics of pemphigus vulgaris (H&E stain) A suprabasal acantholytic blister is seen at low power (A, 40x). A row of intact basal keratinocytes lines the blister floor – producing the characteristic "tombstone" appearance (B, 100x). At higher resolution (C, 200x), acantholysis and a dense mononuclear are seen infiltrating the underlying chorion

The patient maintained follow-up with Internal Medicine and Stomatology in the following months. The biopsy performed during hospitalization showed no acantholysis, and immunofluorescence assays were inconclusive regarding PV. The patient demonstrated a worsening of the oral lesions, which increased in number and size, provoked pain, and caused difficulty feeding, resulting in weight loss. There was a need to increase corticosteroid therapy to 30 mg of prednisolone PO daily. A biopsy of the oral ulcers was repeated, and it showed acantholytic vesiculobullous lesions with an inflammatory infiltrate of mononuclear cells, which is suggestive of PV (Figure 2).

Therefore, the diagnosis of PV was established, and the patient was referred to the Dermatology Clinic. A decision was made to start immunosuppressive therapy with RTX. She completed two infusions of RTX 1000 mg IV within two weeks, after which the oral lesions fully resolved. She has been in complete remission for the past 2.5 years.

## Discussion

PV is a chronic, debilitating dermatologic disease; it has an average age of onset of 40-60 years [5] and is more prevalent among women [8]. Without adequate treatment, PV can be fatal, as extensive skin involvement can result in loss of epidermal barrier function, leading to loss of body fluids, malnutrition, and secondary infections [9]. Even in the earlier stages of the disease and even without extensive skin involvement, PV is associated with a significant impact and high morbidity. As with the case presented here, where the disease manifested only in the oral mucosa, the painful and extensive blistering made feeding a complicated and painful task, leading to food avoidance, malnutrition, significant weight loss, asthenia, and impairment of the general status. The loss of mucosal integrity promoted secondary bacterial infections of the ulcers, which had to be adequately and timely addressed to prevent further complications.

It is not uncommon for the oral mucosa to be the first site affected by PV, as oral lesions are the first manifestation in 50-70% of cases and occur in 90% of patients [10]. Patients can then progress to developing lesions in the skin and other mucosae, but oral involvement may also be the only manifestation of the disease. This pattern of disease reflects the differential tissue expression of desmoglein isoforms, compensatory mechanisms, and the specificity of the autoantibody response [9]. In the oral mucosa, desmoglein 3 (Dsg3) is highly expressed and functions as the primary adhesion protein, whereas in the skin, both desmoglein 1 (Dsg1) and Dsg3 are expressed in distinct layers. When autoantibodies are directed solely against Dsg3, as in the early stages of PV, the oral mucosa is affected first, while the skin is spared due to compensation by Dsg1. As the disease progresses and autoantibodies against Dsg1 develop, cutaneous involvement typically ensues [9]. In cases where oral lesions are the only manifestation, establishing a PV diagnosis may be difficult, as PV oral erosions may mimic other local and systemic diseases, such as aphthous stomatitis, acute herpetic stomatitis, Stevens-Johnson syndrome, Behçet’s disease, and paraneoplastic pemphigus, among others [10].

Indeed, as with this case, the differential diagnosis of oral ulcerations led us to the diagnosis of HSV-1 infection. There was no previous history of HSV-1 infection in our patient, although a significant portion of primary infections is asymptomatic. The fact that IgM and IgG antibodies were detected in blood was not helpful to discriminate between primary and recurrent infection, as IgM antibody titers can increase during recurrence [11]. However, recurrent infections are usually less severe, confined to the lips and perioral region, and are rarely associated with systemic symptoms. Primary oral НSV-1 infection in adults often presents as severe pharyngitis, but up to one-third of the patients present with oral exudative and ulcerative lesions and systemic symptoms [12], which better fits the clinical presentation of our patient.

In the absence of antiviral therapy, gingivοѕtоmаtitiѕ lesions associated with primary НЅV-1 infection last between one to three weeks. However, our patient reported the existence of oral lesions five weeks before inpatient admission and the diagnosis of herpetic gingivοѕtоmаtitiѕ. It is, nevertheless, known that HSV-1 can cause severe and prolonged disease in special populations. Risk factors for severe disease include ΗІV infection, malignancy, organ and hematopoietic stem cell transplantation, malnutrition, pregnancy, and advanced age [13]. Patients with burns and other skin disorders, such as eczema and PV, are also at increased risk for severe HЅV infection [14]. It is likely that malnutrition resulting from the feeding difficulties associated with some degree of immune dysfunction in a patient with an autoimmune background, as evidenced by the previous history of autoimmune gastritis and vitiligo, contributed to the perpetuation of HSV-1 infection.

Another interesting question raised by this case is whether the HSV-1 infection was an accidental finding in the disease course of our patient or if it acted as a trigger for the development of the PV. Viruses have been considered as major environmental factors that trigger autoimmune phenomena in genetically susceptible individuals [15] through mechanisms of molecular mimicry. Herpes viruses have been associated with the pathogenesis of multiple sclerosis, systemic lupus erythematosus, and Sjögren's syndrome [15]. HSV-1 infections are common among patients with PV, often as a treatment side effect, and HSV-1 infection has been associated with worsening of symptoms, treatment resistance, and recalcitrant PV. HSV-1 can thus influence the disease course, but its function as a trigger is not as clear, as several small studies yielded conflicting results [16,17]. However, a recent large population retrospective cohort study suggested that HSV-1 infection is a rare but possible risk factor for PV, shedding some light on the latter [18].

Another factor contributing to the challenge in accurately diagnosing and treating our patient was that the initial biopsy failed to establish the diagnosis. However, it is not uncommon that a biopsy of oral erosions does not yield a diagnosis. Direct immunofluorescence assays are the most accurate in diagnosing mucosal PV [10]. Still, tissue representativity plays a significant factor, and when such techniques are unavailable or fail to provide a diagnosis, repeating the biopsy might be an appropriate course of action, as demonstrated in this case. While awaiting a definitive diagnosis, measurement of anti-desmosomal antibodies may aid in establishing a presumptive diagnosis of PV and in guiding initial treatment [7,9]. However, it is important to note that anti-desmosomal antibodies can be positive even in the absence of clinical or laboratory evidence of disease, and they do not differentiate between PV and pemphigus foliaceus.

Treatment of PV can be challenging as well. The mainstay of the management of the disease is systemic glucocorticoids such as prednisolone, usually 0.5-1.5 mg/kg PO per day [6,7], but severe presentations might require pulsing. Adjunct corticoid-sparing therapies are usually needed to induce and maintain remission and avoid steroid‐related adverse events [6,7]. Such therapies include azathioprine, mycophenolate, and cyclophosphamide [5-7], with varying degrees of success and different adverse reaction profiles. 

RTX has emerged as a promising treatment for PV based on the fact that PV is an antibody-mediated autoimmune disease. By depleting CD20-expressing B cells, RTX treatment can efficiently control the disease, particularly in refractory illness [6,7]. Its cost is still a significant hurdle for its generalized use for PV. It can be used as a first-line maintenance treatment for pemphigus, profoundly impacting disease management. However, no consensus exists on the RTX dosing regimen best suited for PV. Induction treatment of our patient was performed according to the American and European guidelines, which recommend an induction regimen consisting of two infusions of 1000 mg RTX at a two-week interval with a tapering course of glucocorticoids [6,7], and the patient had a complete remission of her disease. Regarding maintenance therapy, the guidelines differ, with even some experts suggesting lower-dose RTX regimens [19] or re-treatment upon relapse [20]. In our patient, a decision for watchful waiting was made, and she has been in remission for the past 2.5 years.

## Conclusions

PV is a rare autoimmune blistering disorder of the skin and mucosa. Blistering of the oral mucosa is frequent in these patients and can be the only manifestation of the disease, posing a challenge in terms of diagnosis in such situations. Establishing a diagnosis might require the exclusion of other blistering disorders and persistence in obtaining histopathological confirmation. Treatment can be challenging as well, as some patients might become corticoid-dependent. RTX has emerged as a safe and very effective medication, both as a first-line treatment and as an add-on approach to induce remission.

## References

[1] Mustafa MB, Porter SR, Smoller BR, Sitaru C (2015). Oral mucosal manifestations of autoimmune skin diseases. Autoimmun Rev.

[2] Ahmed AR, Moy R (1982). Death in pemphigus. J Am Acad Dermatol.

[3] Ding X, Diaz LA, Fairley JA, Giudice GJ, Liu Z (1999). The anti-desmoglein 1 autoantibodies in pemphigus vulgaris sera are pathogenic. J Invest Dermatol.

[4] Seshadri D, Kumaran MS, Kanwar AJ (2013). Acantholysis revisited: back to basics. Indian J Dermatol Venereol Leprol.

[5] Geng RS, Sibbald RG (2025). Pemphigus vulgaris: clinical aspects and treatments. Adv Skin Wound Care.

[6] Joly P, Horvath B, Patsatsi Α (2020). Updated S2K guidelines on the management of pemphigus vulgaris and foliaceus initiated by the European Academy of Dermatology and Venereology (EADV). J Eur Acad Dermatol Venereol.

[7] Murrell DF, Peña S, Joly P (2020). Diagnosis and management of pemphigus: recommendations of an international panel of experts. J Am Acad Dermatol.

[8] Rosi-Schumacher M, Baker J, Waris J, Seiffert-Sinha K, Sinha AA (2023). Worldwide epidemiologic factors in pemphigus vulgaris and bullous pemphigoid. Front Immunol.

[9] Porro AM, Seque CA, Ferreira MC, Enokihara MM (2019). Pemphigus vulgaris. An Bras Dermatol.

[10] Wojnarowska F, Venning VA, Burge SM (2004). Immunobullous diseases. Rook's Textbook of Dermatology.

[11] (2024). American Sexual Health Association: herpes testing. Herpes Testing [Internet.

[12] Annunziato PW, Gershon A (1996). Herpes simplex virus infections. Pediatr Rev.

[13] Piperi E, Papadopoulou E, Georgaki M, Dovrat S, Bar Illan M, Nikitakis NG, Yarom N (2024). Management of oral herpes simplex virus infections: the problem of resistance. A narrative review. Oral Dis.

[14] Keane JT, Malkinson FD, Bryant J, Levin S (1976). Herpesvirus hominis hepatitis and disseminated intravascular coagulation. Occurrence in an adult with pemphigus vulgaris. Arch Intern Med.

[15] Arleevskaya MI, Manukyan G, Inoue R, Aminov R (2017). Editorial: microbial and environmental factors in autoimmune and inflammatory diseases. Front Immunol.

[16] Marzano AV, Tourlaki A, Merlo V, Spinelli D, Venegoni L, Crosti C (2009). Herpes simplex virus infection and pemphigus. Int J Immunopathol Pharmacol.

[17] Vega-Memíje ME, García-Vázquez FJ, Cuevas-González JC, Rodríguez-Lobato E, Aguilar-Urbano MA (2015). Is there a causal relationship between HSV-1 and pemphigus vulgaris?. Springerplus.

[18] Kasperkiewicz M, Ngo BT, Hernandez G, Kridin K, Ludwig RJ (2024). Pemphigus following herpes simplex infection: a global comprehensive cohort study. J Am Acad Dermatol.

[19] Ciolfi C, Tartaglia J, Alaibac M (2024). Is it time to reconsider rituximab dosing regimens for pemphigus vulgaris?. Antibodies (Basel).

[20] Aryanian Z, Balighi K, Emadi SN, Hatami P (2024). Rituximab as a maintenance treatment in patients with pemphigus vulgaris: when is the right time for discontinuation?. J Cosmet Dermatol.

